# Standard internal limiting membrane peeling versus internal limiting membrane abrasion technique for primary epiretinal membrane surgery

**DOI:** 10.5935/0004-2749.2021-0208

**Published:** 2022-07-04

**Authors:** Fatma Bagci, Mehmet Citirik, Selda Çelik Dülger, Mehmet Yasin Teke

**Affiliations:** 1. University of Health Sciences, Ulucanlar Eye Training and Research Hospital, Altindag, Ankara, Turkey

**Keywords:** Epiretinal membrane, Vitrectomy, Diamond-dusted membrane scraper, Membrana epirretiniana, Vitrectomia, Raspador de membrana com pó, de diamante

## Abstract

**Purposes:**

The purpose of this study is to compare the standard inner limiting membrane peeling technique to the inner limiting membrane abrasion technique with respect to visual outcomes and central retinal thickness in the primary epiretinal membrane surgery.

**Methods:**

A total of 59 eyes from 57 epiretinal membrane patients were separated into two groups including the standard inner limiting membrane peeling group and the inner limiting membrane peeling with abrasion technique group. At 6, 12, and 24 months of follow-up, the mean alteration in best-corrected visual acuity and central retinal thickness were assessed for each group.

**Results:**

The study includes 32 (54%) standard inner peeling and 27 (46%) inner limiting membrane peeling with abrasion technique patients. The mean preoperative logMAR best-corrected visual acuity for the standard inner limiting membrane peeling and inner limiting membrane peeling with abrasion groups was 0.73 (±0.29) and 0.61 (±0.3) respectively. At 6, 12, and 24 months of follow-up, the best-corrected visual acuity improved significantly in each group. At each period of observation, the alteration in best-corrected visual acuity was not statistically significant (p=0.54, p=0.52, p=0.67). When comparing the alterations between the standard inner limiting membrane peeling and inner limiting membrane peeling with abrasion technique groups at 6 months (p=0.26) and 24 months (p=0.06), no statistically significant differences were observed, but they were statistically different at 12 months (p=0.03), reflecting a greater reduction in central retinal thickness for the inner limiting membrane peeling with abrasion technique group after one year.

**Conclusion:**

Abrasion of the inner limiting membrane with a diamond-dusted membrane scraper during epiretinal membrane surgery demonstrates similar effectiveness to the standard inner limiting membrane peeling technique. At 12 months, retinal thinning was found to be more significant in inner limiting membrane peeling with abrasion technique patients in terms of central retinal thickness values. As a result, it may be argued that the inner limiting membrane abrasion technique eliminates the inner limiting membrane and related structures more effectively while inflicting less retinal damage.

## Abstract (pt)

**Objetivo:**

Este estudo tem como objetivo comparar a técnica padrão de peeling da membrana limitadora interna com a técnica de abrasão da membrana limitadora interna com relação aos resultados visuais e à espessura central da retina na cirurgia primária de membrana epirretiniana.

**Métodos:**

Cinquenta e nove olhos de 57 pacientes com membrana epirretiniana foram divididos em dois grupos, incluindo o grupo de remoção padrão da membrana limitante interna e o grupo de remoção da membrana limitante interna com técnica de abrasão. A alteração média da melhor acuidade visual corrigida e da espessura central da retina foram medidas para cada grupo aos 6, 12 e 24 meses de acompanhamento.

**Resultados:**

O estudo incluiu 32 (54%) de padrão de membrana limitante e 27 (46%) de membrana interna com técnica de abrasão. A média de logMar pré-operatório de melhor acuidade visual corrigida foi de 0,73 (±0,29) e 0,61 (±0,3) para os grupos de remoção padrão da membrana limitante interna e de remoção da membrana limitante interna com técnica de abrasão, respectivamente. A melhor acuidade visual corrigida melhorou significativamente em cada grupo aos 6, 12 e 24 meses de acompanhamento. A alteração na melhor acuidade visual corrigida não foi estatisticamente significante (p=0,54, p=0,52, p=0,67) em cada período de observação. Quanto à espessura central da retina, diferenças estatisticamente significativas não foram observadas aos 6 meses (p=0,26) e 24 meses (p=0,06), mas foram estatisticamente diferentes aos 12 meses (p=0,03) quando comparadas às alterações entre os grupos de remoção padrão da membrana limitante interna e de remoção da membrana limitante interna com técnica de abrasão, refletindo uma maior redução da espessura central da retina para o grupo de remoção da membrana limitante interna com técnica de abrasão após um ano.

**Conclusão:**

A abrasão da membrana limitante interna com um raspador de membrana com pó de diamante em cirurgia de membrana epirretiniana demonstra eficácia semelhante com a técnica de remoção padrão de membrana limitante interna. Em relação aos valores de espessura central da retina, o afinamento da retina foi mais significativo em pacientes com remoção da membrana limitante interna com técnica de abrasão aos 12 meses. Assim, pode-se argumentar que a técnica de abrasão da membrana limitante interna remove a membrana limitante interna e as estruturas relacionadas de forma mais eficaz sem causar danos significativos à retina.

## INTRODUCTION

Epiretinal membrane (ERM) is a common vitreoretinal interface disease that manifests itself as a fibrocellular structure on the inner surface of the neurosensorial retina. It is mainly found in elderly patients without any identifiable cause and is referred to as the idiopathic form. It can, however, develop secondary to ocular or systemic diseases or retinal injuries including retinal vascular disorders, proliferative diabetic retinopathy, retinal breaks, inflammation, retinal laser treatment, and intraocular surgery^(1)^. The prevalence of ERMs is reported to be between 3.5% and 6.9%^(2)^.

The most common symptoms of ERM are reduced and distorted central vision caused by the distortion of normal retinal structure and layers induced by membrane contraction. Pars plana vitrectomy (PPV) and ERM removal have long been used in the treatment of patients with symptomatic ERM as safe and effective procedures with favorable visual outcomes^(3^,^4)^. However, recurrence of ERM was reported in around 10% of patients who underwent surgery^(5)^. Thus, to minimize the necessity of reoperation due to the recurrences, inner limiting membrane peeling has been performed as an additional surgical step in the course of PPV and ERM removal^(6)^.

Favorable outcomes of inner limiting membrane (ILM) peeling for ERM surgery led to its frequent use and adoption as an almost routine practice^(6^,^7)^. Contrary to potential benefits, a growing number of studies reported some deleterious results such as functional and mechanical damage to the retina after the removal of ILM^(8^,^9)^. While there are controversies in the literature on the visual outcomes^(10)^, safety, and indications for ILM peeling in patients with ERM, there is an agreement on the impact of ILM peeling on minimizing recurrences^(11)^. As a result, ILM peeling is likely to remain a standard procedure in ERM surgery. However, to better understand the contradictory conclusions regarding the effectiveness of ILM peeling, the efficacy of evolving alternatives to conventional peelings must be evaluated.

In this study, the central retinal thickness (CRT) and visual outcomes of ERM surgery were evaluated using the standard ILM peeling technique and the ILM abrasion technique (using a diamond-dusted membrane scraper).

## METHODS

### Study design

This single-center retrospective study analyzed the medical records of 59 eyes of 57 consecutive patients with primary (idiopathic) ERM. All patients underwent PPV by two vitreoretinal surgeons (MYT and MC) at the Ulucanlar Eye Training and Research Hospital, Ankara, Turkey, between January 2017 and December 2019. The subjects who were taking any medication, had >6 diopter myopia, or had a history of systemic and ocular disease were excluded from the study. None of the patients reported a history of diabetes mellitus, hypertension, connective tissue diseases, malignancies, or other systemic disorder, nor had any undergone prior vitreoretinal surgery, ocular trauma, or any corneal pathology. Patients were divided into two groups: the standard ILM peeling group (SIP) and ILM peeling with abrasion technique group (AIP). Cases were selected consecutively; while one of the surgeons (MÇ) performed the AIP technique, the other (MYT) performed the SIP technique. The OCTs were examined by a retina specialist who was blinded to the patient and visual acuity outcomes. Retinal architecture and CRT measurements were evaluated with the OCT. This study followed the tenets of the Declaration of Helsinki and the study protocol was approved by Yıldırım Beyazıt Dışkapı Training and Research Hospital Ethical Committee.

### Surgical technique

All surgeries were performed under local anesthesia. The 25-gauge Constellation System (Alcon Laboratories, Fort Worth, TX, USA) was used in all cases. The technique included the insertion of a cannula using a beveled trocar, following the displacement of the conjunctiva to purposefully misalign the conjunctival and scleral incisions with oblique entries. Transscleral cannulas were inserted through the pars plana. In cases with moderate cataracts, phacoemulsification (PE) and intraocular lens (IOL) implantation were performed before vitrectomy through a 2.2-mm clear corneal incision. Hydrophobic acrylic monofocal IOLs (Acrysof IQ SN60WF, Alcon Laboratories Inc.) were inserted into the capsular bag in all cases, followed by stromal hydration application to the corneal wound.

All cases underwent core vitrectomy, followed by the removal of the posterior hyaloid membrane and vitreous traction. The ERM was removed with the Grieshaber DSP 25-G end-gripping forceps (Schaffhausen, Switzerland) with the help of trypan blue. ILM was stained with the brilliant blue G (Dorc International, Zuidland, The Netherlands) in all cases. The dye was injected gently over the macular region, while the infusion was temporarily discontinued. After 30 s, the infusion was restarted and the dye was aspirated by using a vitrectomy probe. In the SIP technique, the ILM was peeled by the pinch technique with the Grieshaber DSP 25-G end-gripping forceps (Schaffhausen, Switzerland) and a peel radius of approximately two-disc diameters. In case of incomplete staining of the ILM with adherent pre-ILM tissue, a normally stained area of ILM was selected from where the peeling was initiated. The ILM was hence peeled en bloc.

**Figure 1:**
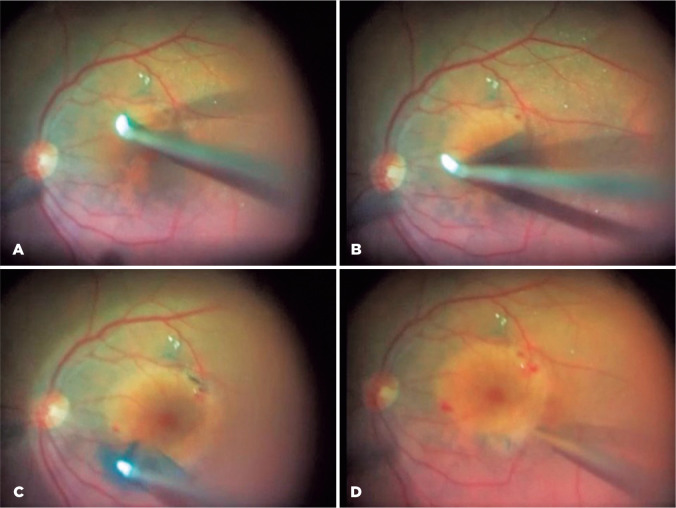
Abrasion technique with a diamond-dusted membrane scraper. (A) Flap was created and folded back on itself. (B) The peeling area was extended in the counter-clockwise direction. (C) The remainig ILM on the foveal region was peeled off by pulling the peeled membranes with DDMS. (D) The peeling was completed with the abrasion technique.

In the AIP technique, diamond-dusted membrane scraper (DDMS) was applied to initiate ILM peeling and remove it completely. First, a flap was created inferior to the fovea at a distance of approximately two-disc diameter from the foveal center through repeated brush motion with DDMS. Second, the ILM flap was folded back on itself (Figure 1A). With consequent brushing motion restricted to the everted flap only, the peeling area was extended in a counter-clockwise direction (Figure 1B). Shearing was continued in a circular motion until completed, similar to that while performing capsulorhexis in cataract surgery. Finally, the remaining ILM on the foveal region was peeled off by pulling the peeled membranes with DDMS (Figure 1C, D). Then, the air-fluid exchange was performed in all cases. Finally, surgery was completed by the removal of the entry site alignment cannulas without the suture of the conjunctiva and sclera.

The complete ophthalmological examination including best-corrected visual acuity (BCVA), intraocular pressure (IOP) with applanation tonometry, slit-lamp biomicroscopy, and dilated fundus examination was performed before the surgical intervention. BCVA was measured using the Snellen chart. The Snellen values were converted to the logMAR for statistical analyses. The IOP was measured with the Goldmann applanation tonometer. The ERM was evaluated by using spectral-domain optical coherence tomography (SD-OCT). An SD-OCT volume scan (20 × 20 with 49 horizontal sections, ART 15) including en face images and macular mapping image obtained with HRA2 (Heidelberg Retina Angiograph-Optical Coherence Tomography, Heidelberg Engineering, Heidelberg, Germany) of the macula was performed. Postoperatively, the data were collected at 6, 12, and 24 months and then at the last visit after surgery. The mean change in distance BCVA (logMAR) and CRT were measured for each group at 6-, 12-, and 24- month follow-ups.

### Primary and secondary outcome measures

The primary outcome measure was the mean change in BCVA and CRT at a 6-month follow-up for each group. The secondary outcome measures included the mean change for each treatment group in the BCVA change at 12 and 24 months and the change in CRT by the SD-OCT at 12 and 24 months. CRT was defined as the highest value on the standard Spectralis SD-OCT retinal thickness map.

### Statistical analyses

Descriptive and statistical analysis was performed using the STATA 15 software. Skewness/Kurtosis tests for normality were conducted for both the groups at each period of observation, and the test results did not reject the hypothesis that the samples were normally distributed at a 5% confidence level. Therefore, a comparison of the statistical differences between the two groups (mean differences) was performed by a two-sample t-test as per the standard methodology. Preoperative and postoperative variables were presented in terms of the mean and standard deviation. P<0.05 was considered to indicate statistical significance.

## RESULTS

### General characteristics of the sample

The research compromised 59 eyes from 57 patients who underwent ERM surgery with ILM peeling. There were 27 patients (46%) in the AIP group, 13 of whom were male, and 32 patients (54%) in the SIP group, 14 of whom were male. The patients evaluated had a mean age of 67 (±7) in the AIP group and 66 (±8) in the SIP group. The minimum follow-up period was 2 years. The 25-gauge system was used in all patients.

### Main outcomes

The mean changes in preoperative and postoperative measurements are compared between eyes that had SIP and those that received ILM peeling with abrasion technique (AIP). At 6, 12, and 24 months of follow-up, the mean change in BCVA (logMAR) and CRT were mea­sured for each group.

The mean preoperative logMAR visual acuity for the SIP and ILM peeling with abrasion groups was 0.73 (±0.29) and 0.61 (±0.3), respectively. BCVA improved significantly in each group after 6, 12, and 24 months of follow-up. The mean change in BCVA at 6, 12, and 24 months was 0.49 (±0.3), 0.43 (±0.31), 0.43 (±0.28) logMAR in the SIP group and 0.42 (±0.26), 0.36 (±0.24), 0.34 (±0.25) logMAR in the AIP group, respectively (Table 1). Similarly, both groups showed significant improvement in CRT, particularly at 6 and 12 months. The mean values of CRT at 6, 12, and 24 months were 386 (±71), 345 (±63), and 337 (±39) in the AIP group and 401 (±68), 383 (±64), and 366 (±58) in the SIP group. The indices in both groups show a clear improvement over time.

**Table 1:** Comparison of the mean and SD values of BCVA and CRT (the baseline values and the ones at 6, 12, and 24 months

		**AIP**	**SIP**	**p-value**
PREOP	BCVA, logMAR ± SD,	0.61 (±0.3)	0.73 (±0.29)	
	Central retinal thickness (CRT), µm (SD)	488 (±80)	477 (±99)	
AT 6M	BCVA, logMAR ±SD,	0.42 (±0.26)	0.49 (±0.3)	0.536
	Central retinal thickness (CRT), µm (SD)	386 (±71)	401 (±68)	0.263
AT 12M	BCVA, logMAR ±SD,	0.36 (±0.24)	0.43 (±0.31)	0.520
	Central retinal thickness (CRT), µm (SD)	345 (±63)	383 (±64)	0.030
AT 24M	BCVA, logMAR ±SD,	0.34 (±0.25)	0.43 (±0.28)	0.673
	Central retinal thickness (CRT), µm (SD)	337 (±39)	366 (±58)	0.064

**Figure 2:**
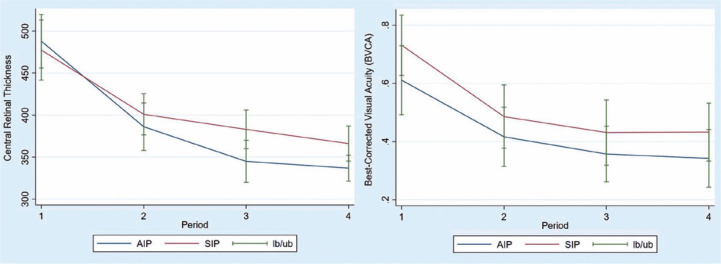
Mean change in the central retinal thickness (CRT) and best-corrected visual acuity (BCVA) in the AIP and SIP groups was measured for each patient as the difference from the preoperative value (period 1). Error bars represent the lower bound (LB) and upper bound (UB) for standard error of the mean value. Period 2 refers to follow-up at 6^th^ month, period 3 refers to follow-up at 12^th^ month, and period 4 refers to follow-up at 24^th^ month.

Table 1 includes two-sample t-test results to check if there is a statistically significant difference in the improvements observed in both groups. The changes in BCVA observed in both groups were not statistically significant (p=0.54, p=0.52, p=0.67) at each period of observation. In terms of CRT, no statistically significant differences were observed at 6 months (p=0.26) or 24 months (p=0.06), but they were statistically different at 12 months (p=0.03), indicating potentially more favorable results for AIP after one year of the operation. The graphical representation also indicates that CRT values in the AIP group fall at a higher rate than the CRT values in the SIP group (Figure 2). As demonstrated in the Figure, there are no obvious differences in the pattern of BCVA values of both comparison groups.

**Figure 3:**
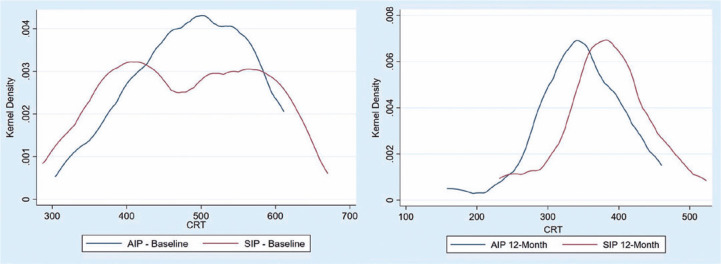
Distribution of preoperative and postoperative values (at 12^th^ month) of central retinal thickness (CRT) in the AIP and SIP groups.

Moreover, Figure 3 demonstrates that, while the distribution of the preoperative CRT values in both samples was similar, the distribution of the postoperative values at the 12^th^ month was leftward-sided, indicating that patients who underwent ERM surgery with ILM peeling by using abrasion technique achieved better results.

## DISCUSSION

The ILM, a transparent structure that establishes the boundary between the retina and the vitreous, may be critical in explaining the pathophysiology of retinal disorders involving the vitreomacular interface. Due to its contiguous relations with Muller cells and ERM, it acts as a rigid scaffold that transmits the distortion caused by the ERM onto the more flexible underlying retina. Due to the advantages, it offered to surgeons, ILM peeling has become a popular surgical approach in ERM removal procedures^(12)^. These include favorable surgical results in achieving closure of macular holes^(13^,^14)^, increasing evidence on the safety of vital dyes (indocyanine green dye and brilliant blue G) usage in vitreoretinal surgeries, and reduced ERM recurrences as a result of complete removal of ERM by peeling the ILM^(11)^.

Considering its widespread usage, there have been a plethora of studies on the impacts of ILM peeling on visual outcomes, anatomical changes such as CRT, and more recently retinal functional consequences. Concerning BCVA, it has long been established that ILM peeling during ERM surgeries does not result in additional postoperative improvement in visual outcomes^(15)^. This is supported by recent studies investigating the long-term impacts extending to 3-5 years^(16)^. With regards to CRT, there are contradictory findings in studies investigating short-term consequences for up to 1 year. A recent study investigating long-term effects found an initially progressive decline in postoperative retinal thickness, but no significant effect after 5 years^(16)^. Concerning retinal functional consequences, a growing number of studies have begun to evaluate the subclinical influence of ILM peeling to investigate possible mechanical damage to the retina using tools such as microperimetry^(17)^, multifocal electroretinography^(18)^, or the Humphrey perimeter^(19)^. A meta-analysis interpreted that the findings were restricted to subclinical levels due to subtle retinal damage^(11)^.

Thus, in the absence of surgical complications, ILM peeling in ERM surgery is crucial for avoiding recurrences. However, complications including macular holes, visual field defects, and photoreceptor dysfunction can occur due to either surgical technique or intraoperative problems^(15^,^20)^. These adverse events have been well described and must always be taken into account when determining overall surgical outcomes^(12)^.

Several surgical techniques, adjuvants, and equipment have been introduced to identify the ILM and facilitate its peeling without causing collateral retinal damage. However, none of them have been proven to be superior in terms of causing minimal retinal damage. The creation of an ILM flap is a critical step in these techniques for allowing the peel to be initiated^(21)^. Three common approaches to creating a flap are (i) the direct “pinch” technique using custom-designed forceps, preferred by many surgeons (ii) creating a defect in ILM by picks and microvitreoretinal blades, and (iii) using a diamond-dusted sweeper (Dorc) or sweep of a DDMS across the ILM surface (called as abrasion technique) or recently a micro-serrated nitinol loop (Finesse Flex Loop, Alcon, Ft. Worth, TX). In this study, a 25 gauge extendible diamond-dusted sweeper was employed (Dorc, Dutch).

The CRT and visual outcomes of the SIP technique (direct pinch technique) in ERM surgery are compared in this study to the ILM abrasion technique utilizing a diamond-dusted sweeper.

Only a few studies have reported on the outcomes of the ILM abrasion technique used mainly in cases with macular holes. According to Mahajan et al., a 94% success rate in successfully closing MHs using the ILM abrasion technique is equivalent to the rates in MH surgeries utilizing the conventional ILM peeling technique^(22)^. They employed triamcinolone as a stain to limit the risk of dye toxicity, which might lead to reduced visual acuity outcomes, especially regarding indocyanine green^(23)^. Using vital dyes, on the other hand, provides a distinct contrast between the ILM and deeper retinal tissue, facilitating the stripping of all epiretinal tissue, including the ERM and ILM, without having to worry about peeling the tissue from the underlying retina. Thus, once triamcinolone is used, it will be more difficult to visualize the ILM, with a higher risk of retinal injury. Moreover, many well-executed studies provided evidence that brilliant blue G is a safe and ideal dye for ILM due to its affinity, reduced toxic profile, and ability to minimize the appea­rance of apoptosis^(10^,^24^,^25)^. To address these problems, brilliant blue was employed in both the SIP and AIP groups in the current investigation.

The study by Mahajan et al.^(22)^ entailed several shortcomings, including varied follow-up durations (in some cases as short as 3 months) and a lack of a control group for comparison with SIP. Steel et al. found less dissociated optic nerve fiber layer appearance (DONFL, a distinctive change in the appearance of the inner retina)^(26)^ on SD-OCT and retinal debris on transmission electron microscopy with forceps peeling compared to abrasion technique by DDMS^(27)^ when investigating the effect of abrasion and SIP techniques in surgeries for idiopathic macular holes.

Finally, Almeida et al. described an ILM abrasion technique to address elements of tangential traction on the retinal surface to achieve successful macular hole closure without complete removal of the ILM as well as to limit the loss of adjacent tissue of the inner retina and eliminate the risk of adjuvant dye toxicity^(28)^. They studied three donor eyes with macular holes to identify the effect of various tactile pressures (i.e., none, light, medium, and heavy) applied with a 23-gauge DDMS on the retinal surface. Comparing the outcomes of these tactile pressures, they found no disruption of the RNFL or deeper retinal layers, indicating that the DDMS may only remove the surface layer of ILM without penetrating the RNFL, contrary to previous findings on entire peeling of ILM by applying heavier pressure on the retinal surface^(29^,^30)^.

This study adds to the literature by providing new evidence on the potential benefits of this ILM abrasion technique versus SIP by addressing some of the shortcomings of previous studies, particularly in terms of longer follow-up periods (minimum 2 years) and a control group of SIP. At 6, 12, and 24 months of follow-up, both SIP, and ILM abrasion groups showed similar levels of improvements in BCVA. However, no statistically significant differences in CRT, were observed at 6 or 24 months, but they were statistically different at 12 months when comparing the changes between the SIP and AIP groups, reflecting a greater reduction in CFT for the AIP group. The possible concern at this point is that diamond-dusted membrane scraping may induce potential iatrogenic damage to the inner retinal layers via the ILM abrasion technique. Following such damage, it may manifest as atrophy on OCT and a decrease in CRT. In this study, the diamond-dusted membrane scraping was used by an experienced surgeon, and a postoperative OCT examination revealed no signs of atrophy caused by the use of an instrument. Moreover, the disparity in CRT values between the two groups reduced after 24 months. As a result, it may be argued that the ILM abrasion technique removes ILM and related structures more effectively without causing significant retinal damage. In this connection, additional studies need to be conducted by taking into consideration the various types of ILM peeling techniques, surgeon learning curve, and use of dye to generate further evidence on the potential benefits of alternative ILM techniques vis-à-vis standard ILM techniques.

Since each surgeon performed only one technique, a drawback of our study is that surgeon-specific differences and experience factors, rather than the peeling technique itself, may have resulted in the differences obser­ved between the two techniques. To address this concern, future studies may compare the results of surgeries conducted by a single surgeon using both techniques.

Another limitation is that the study did not include a scoring system to quantify the observable petechial hemorrhages following ILM separation from the retinal surface, especially when creating an ILM flap, which is a critical step in allowing the peel to be initiated. Despite the absence of a scoring system, we observed some petechial retinal hemorrhages caused by the ILM separating from the underlying retina in both techniques, although they were more superficial and of lesser magnitude in the abrasion technique, reflecting lower disruption of the nerve fiber layer (NFL). However, future studies are needed to provide further evidence, particularly on the potential advantages of the abrasion technique in patients with thin NFLs, such as those with advanced glaucoma, where potential disruption of the NFL might be detrimental.

Abrasion of the ILM using a diamond-dusted membrane scraper during ERM surgery is as successful as the SIP technique. After one-year, retinal thinning was found to be more significant in AIP patients in terms of CRT values. Even if the difference between the two groups becomes statistically insignificant after 24 months, average CRT outcomes remain comparably lower. As a result, it may be argued that the ILM abrasion technique eliminates ILM and its associated structures more effectively without causing significant retinal damage.
